# Making sense of vitamin D concentrations

**DOI:** 10.4155/fso.15.90

**Published:** 2016-01-13

**Authors:** Florin M Musteata

**Affiliations:** 1 Department of Pharmaceutical Sciences, Albany College of Pharmacy & Health Sciences, 106 New Scotland Avenue, Albany, NY 12208, USA

**Keywords:** 1,25-dihydroxyvitamin D, 25-hydroxyvitamin D, bioavailable concentration, free concentration, normalized concentration, total concentration, vitamin D

Hardly a day passes without a scientific or news article touting the benefits of vitamin D supplementation, only to be followed shortly by articles cautioning about the lack of benefits or even the severe adverse effects of vitamin D compounds. The most discussed articles are those investigating the link between vitamin D status and diseases such as dementia [1,2] or cancer [3,4], although articles about the influence of vitamin D on infections, asthma, anorexia, cardiovascular diseases, diabetes, and more than a hundred other outcomes [5] are just as common. The main recognized function of vitamin D is to maintain appropriate levels of calcium and phosphate for bone mineralization, muscle contraction, nerve conduction and general cellular function [6]. Nevertheless, recent evidence has shown that the active metabolites of vitamin D may also be able to regulate different cellular processes associated with carcinogenesis [7]. Furthermore, maintaining an optimal level of vitamin D may help in preventing the risk of obesity, mental disease, depression and asthma [1,8,9].

The core reason for the great interest in vitamin D compounds is the recent discovery that almost all mammalian cells contain the vitamin D receptor and nearly 3% of the human genome is regulated by a vitamin D-related pathway [6]. Much of the controversy surrounding vitamin D studies is linked to the great complexity surrounding its sources, metabolism and action in the body. Vitamin D is a family of substances that contains more than 40 different compounds [7]. While the D_3_ form can be produced naturally in the human skin by exposure to UV light, two forms (vitamin D_2_ and D_3_) with somewhat different potency can be found naturally or fortified in various foods. A further complication is the fact that D_2_ and D_3_ are not active but have to go through several metabolic steps for activation.

To make investigations of health outcomes even more perplexing, the form that is recommended for monitoring vitamin D status is neither the parent vitamin D nor the active compound (1,25-dihydroxyvitamin D) but an intermediary metabolite (25-hydroxyvitamin D). Ideally, a full study of the relationships between vitamin D and health outcomes should include long-term monitoring of all vitamin D forms: dietary sources, inactive metabolites and active metabolites. However, due to the general lack of suitable analytical methods that can monitor all vitamin D forms as well as the relatively high cost of the methods that can monitor some of the metabolites, most clinical studies are based on monitoring either the intake of vitamin D or the concentration of the inactive metabolite (which has a long half-life, is present at higher concentrations than most of the other compounds and is currently considered the best singular marker of vitamin D status).

Given this amazing complexity of the vitamin D pathways and the great variation in the type of parameters monitored by various studies, it is not surprising that general consensus regarding the importance of vitamin D cannot be found. Currently, the most reliable studies are considered to be those that monitor the concentration of 25-hydroxyvitamin D, the inactive metabolite. Nevertheless, even these studies are surrounded by controversy, since many of the analytical methods on the market have rather high uncertainty and suffer from multiple interferences [8,9].

Another issue with current studies is that they usually only look at one or two concentration data points per study participant; ideally, the full lifetime exposure to vitamin D compounds should be considered. At a minimum, the area under the concentration–time curve over a few years should be measured for investigating the correlation with diseases that take a long time to develop, such as dementia and cancer. Furthermore, all studies should include testing of vitamin D-related genetic variation, which can greatly impact not only the vitamin D levels but also the clinical response to the vitamin. Equally important to consider are the recently discovered autocrine and paracrine vitamin D systems that operate in many tissues; this type of signaling is based on local synthesis and utilization of vitamin D metabolites; deficiencies of this local system may play a significant role in the relationship between vitamin D and cancer [10]. To address the need to investigate the existence and roles of local vitamin D systems, the tissue distribution of these compounds should be determined. Additionally, the existence of tissue-specific vitamin D-binding proteins – if found – might explain the lack of correlation between total plasma levels and clinical effects. Finally, reliable studies should address the issue of reverse causation; in many cases, it might be possible that a certain disease state causes the subjects to spend more time indoors which in turn results in lower vitamin D levels.

One of the main impediments to finding good correlations between vitamin D metabolite concentrations and effects at population levels is the high interindividual variability in vitamin distribution between carrier proteins and target receptors. More than 99% of vitamin D and its circulating metabolites are reversibly bound to various proteins in plasma and tissues. This binding to various proteins changes the relationship between the measured concentration and biological effects [8,10]. Different people have widely different concentrations of binding proteins in plasma [11], and therefore the same concentration of 25(OH)D can produce very different health outcomes. The plasma proteins that vitamin D compounds bind to most commonly are albumin and vitamin D-binding protein, which are known to have significant genetic variation in the population.

The total concentrations of vitamin D metabolites currently measured in clinical practice do not compensate for interindividual variability in distribution, while calculated or measured free concentrations have been recognized to be better correlated with effects to some extent [11,12]. On one hand, there are reasonably accurate and reproducible analytical methods based on measuring total vitamin concentrations that are poorly correlated with health outcomes, and on the other hand, there are less accurate but better correlated methods for measuring or calculating free vitamin concentrations. Accordingly, progress in finding good population-level correlations between vitamin D concentrations and health outcomes has been slow.

To avoid the problems related to using total and free concentrations in clinical studies, other types of concentrations that are better related to the effects of the vitamin have been sought. One such type is the bioavailable vitamin D concentration, representing the vitamin molecules that are not bound to vitamin D-binding protein. The assumption here is that the molecules bound to albumin and those that are free in plasma are available for activity, while those bound to the specific binding protein are not related to activity. Several research papers have shown that the bioavailable concentration is correlated to effects much better than the total concentration and slightly better than the free concentration [11,13]. While the free and bioavailable concentrations are indeed better correlated to clinical outcomes, there are no established reference ranges for them and they are difficult to interpret since their values are three to four orders of magnitude lower than those of the corresponding total concentrations.

A more recent approach to compensate for differences between patients when interpreting laboratory data is based on calculating the normalized concentration of the investigated bioactive compound, based on either the free or total concentration and the overall composition of the investigated organism [14]. In the case of vitamin D compounds, the normalized concentration for a particular patient would be the concentration that produces a similar pharmacodynamic effect in an individual with average body composition in which the normal levels of various markers are usually established [15]. The simplest way to normalize concentrations for a particular individual is based on calculating the total concentration that would generate the same free concentration in an individual with average plasma protein binding. When normalized concentrations were determined based on measured free or total vitamin D metabolite concentrations, statistically significant improvements in the relationship between vitamin D metabolites and several markers of health status were found [15].

To conclude, several types of concentrations are available for vitamin D research. The total concentration is easier to measure accurately while the free concentration, which can either be measured or calculated, is usually better correlated with effects. To improve the correlations even further, more refined calculations can be used to determine bioavailable concentrations and normalized concentrations. While bioavailable concentrations intuitively point to the fraction of vitamin that reaches target tissues, normalized concentrations can be customized for any type of effect and are easier to compare with currently established ranges. For example, if the optimal total concentration of 25-hydroxyvitamin D was established to be around 75 nM in individuals with average body composition, then individuals with different body composition should have their normalized concentration around 75 nM as well in order to experience the maximum benefit from vitamin D.
